# Recombinant KRAS G12D Protein Vaccines Elicit Significant Anti-Tumor Effects in Mouse CT26 Tumor Models

**DOI:** 10.3389/fonc.2020.01326

**Published:** 2020-08-12

**Authors:** Yuhua Wan, Yan Zhang, Gengchong Wang, Patrick Malonza Mwangi, Huaman Cai, Rongxiu Li

**Affiliations:** ^1^State Key Laboratory of Microbial Metabolism, School of Life Sciences and Biotechnology, Shanghai Jiao Tong University, Shanghai, China; ^2^Shanghai HyCharm Inc., Shanghai, China; ^3^Engineering Research Center of Cell and Therapeutic Antibody, Ministry of Education, Shanghai Jiao Tong University, Shanghai, China

**Keywords:** KRAS, diphtheria toxin, vaccine, immune response, G12D

## Abstract

Drug development targeting the most frequently mutation G12D of KRAS has great significance. As an attractive immunotherapy, cancer vaccines can overcome binding difficulties of small molecules; however, the weak immunogenicity and production difficulties of reported KRAS mutation vaccines limit their clinical application. To improve antigen-specific immune responses and Anti-Tumor effects on tumors expressing KRAS G12D mutation, we designed recombinant proteins containing KRAS peptide (amino acids 5–21) with G12D (called SP) in two forms: DTT-SP_4_ and DTSP. DTT-SP_4_ was constructed by fusing four copies of SP to the C-terminal of the translocation domain of diphtheria toxin (DTT), and DTSP was constructed by grafting SP onto DTT. The two vaccines in combination with aluminum hydroxide (Alum) and cytosine phosphoguanine (CpG) successfully induced conspicuous SP-specific humoral and cellular immune responses, and displayed prominent protective and therapeutic Anti-Tumor effects in mouse CT26 tumor models. Surprisingly, the DTSP-treated group displayed better Anti-Tumor effects *in vivo* compared with the DTT-SP_4_-treated and control groups. Moreover, 87.5 and 50% of DTSP-treated mice in the preventive and therapeutic models were tumor free, respectively. Notably, in the DTSP-treated group, the interferon-γ (IFN-γ) expression of T cells *in vitro* and the T-helper 1 (Th1)–related cytokine expression in tumor tissues indicated that the activated Th1 immune response may be involved in Anti-Tumor activity. Furthermore, DTSP treatment remarkably altered the subpopulation of T cells in splenocytes and tumor-infiltrating lymphocytes. The percentage of effector CD8^+^ T cells increased, whereas that of immunosuppressive CD4^+^Foxp3^+^ T cells remained reduced in the DTSP group. Dramatic tumor-inhibitory effects of DTSP, which is easily prepared, make it a more attractive strategy against KRAS G12D tumors.

## Introduction

KRAS mutations, as common driver mutations, are mainly observed in pancreatic cancer (PDA), colorectal cancer (CRC), and lung cancer, with mutation frequencies of 97.7, 44.7, and 30.9%, respectively (1). Mutant KRAS promotes not only the proliferation of cancer cells but also the infiltration of immunosuppressive cells such as regulatory T cells (Tregs) and reduces the proportion of CD8^+^ T cells in tumors (2–6). Genetic aberrations such as KRAS mutations are specific to cancer and do not exist in normal tissues (7). Thus, targeting the typical hot-spot mutations in KRAS is an attractive approach in *KRAS* mutated cancer treatment.

Unfortunately, KRAS was once considered as an “undruggable” target because it lacks hydrophobic pocket for drugs to bind (1, 8, 9). Amgen reported the first-phase clinical effect of a KRAS-G12C inhibitor AMG-150 (NCT03600883), with an effective rate of 54% and a disease control rate of 100% at a high dose (960 mg/day) in 2019 (10). However, for other KRAS mutations, small molecule drugs still remain elusive, with no effective targeted therapy at present for patients with KRAS-mutant cancer (1, 9, 11).

Mutated RAS peptides loaded on antigen-presentation cells can induce RAS mutation-specific T-cell responses (12–15). This reveals that mutant KRAS proteins can be presented on the cell surface through intracellular processing. Activated KRAS mutation-specific T cells can kill KRAS-mutant tumor cells, leading to the inhibition of KRAS-mutant tumor growth. Therefore, KRAS targeting immunotherapy, which can avoid the necessity of binding with KRAS hydrophobic pockets, has attracted attention. Tran et al. have identified cytotoxic T-cell response against KRAS G12D mutation in tumor-infiltrating lymphocytes (TILs) and all seven metastatic lung nodules of the patient carrying G12D mutation were regressed after the expanded TILs infusion (16). Although TILs are difficult to isolate, purify, and prepare on a large scale, this exciting result provides a good evidence for immunotherapy against KRAS mutations.

The use of vaccines, which are an active immunotherapeutic method for KRAS-mutant cancer, can overcome the binding problem of small molecule drugs and the difficulty of isolating and preparing TILs. Mutant KRAS peptides in combination with different adjuvants have been used in a series of clinical trials. Their safeties have been proven (17–20); however, the peptide-specific T-cell response and Anti-Tumor activity have not been confirmed in these studies. Most of the clinical trials reported previously were stopped in phase I/II (17, 19–22). Because of its weak immunogenicity, KRAS-mutant peptide vaccines do not appear to be immunogenic to all patients (23, 24), although they harbor predicted major histocompatibility complex (MHC) I alleles binding to KRAS mutations with considerable affinity (17, 25). The weak or inconclusive immune response induced by reported KRAS vaccines hinders the clinical use of these vaccines. Thus, an effective means for enhancing the immune response of mutant KRAS vaccines is urgently needed.

In this study, we focused on the G12D mutation, which represents the highest frequency of KRAS mutations (26). To enhance the immune response of the mutant KRAS G12D peptide, we fused the mutant peptide SP with a previously reported carrier protein DTT (27) and designed two forms of the peptide vaccine: DTT-SP_4_ and DTSP. We first confirmed humoral and cellular responses induced by the two Alum and CpG formulated vaccines. Subsequently, we tested Anti-Tumor effects of the two vaccines *in vivo* both therapeutically and prophylactically in a mouse CT26 tumor model, wherein the mice contained a KRAS G12D mutation. Both vaccines, and particularly DTSP, showed dramatic Anti-Tumor effects. Further analysis suggested that the Anti-Tumor efficacy of DTT-SP_4_ or DTSP was associated with an enhanced antigen-specific Th1 response and alteration of immunosuppressive Treg cells and effector CD8^+^ T cells in spleens and tumor tissues.

## Materials and Methods

### Cell Lines and Animals

Colon carcinoma cell line (CT26) was obtained from the Chinese Academy of Sciences Cell Bank located in Shanghai, China. Cells were maintained in RPMI-1640 medium (GIBCO) supplemented with 10% (*v*/*v*) fetal bovine serum (FBS; GIBCO) and 1% streptomycin–penicillin (P/S) at 37°C with 5% CO_2_. DH5α cells used for cloning and Rosetta (DE3) cells used for protein production were from our laboratory.

Five-week-old female BALB/c mice were purchased from SLCAS Laboratory Animal Center (Shanghai). The mice were used for experiments after 1 week of adaptive feeding in the animal center of Shanghai Jiao Tong University. All protocols were approved by the animal care committee of Shanghai Jiao Tong University.

### Mutation Verification and Gene Cloning

Total RNA was extracted from CT26 cells using Trizol reagent (QIAGEN, Beijing, China). cDNA was obtained using a Prime Script RT reagent Kit (Takara Biotechnology, China), and subsequently, KRAS full-length (NM_021284.6) gene was amplified from the generated cDNA. After this, the DNA fragment was inserted into a pEGX-6p-1 vector for sequencing to confirm that the CT26 cell line used in this study contained the G12D mutation.

Four repeats of SP (SP_4_) were linked with each other via a glycine linker (GG). DNA sequences encoding for SP_4_, synthesized by Hua Gene Biotechnology (Shanghai, China) were inserted into the plasmid pUC19. KRAS G domain DNA fragments (named FD^mut^) (amino acids 2–164) were amplified from the KRAS full-length gene containing the G12D mutation. The gene encoding for DTT (amino acids 202–378 of the diphtheria toxin) was from our laboratory, and the position 88–94 was used for SP displacement.

To construct expression plasmids of DTT-SP_4_, DTT-FD^mut^, and DTSP, fusion gene products were obtained through the overlapping PCR technique. All fragments were digested by NdeI and XhoI restriction enzymes and cloned into the His-tagged vector pET28a (GE Healthcare). FD^mut^ and SP_4_ were linked to the C-terminal of DTT via GGGGS and GG linkers, respectively.

DTT-FD^wt^ within KRAS wild-type G-domain and DTSP^wt^ within KRAS wild-type peptide (amino acids 5–21) were constructed by site-directed mutagenesis.

### Preparation of Fusion Protein

DTT-SP_4_, DTT-FD^mut^, DTT-FD^wt^, DTSP, DTSP^wt^, and FD^mut^ expression vectors were transformed into *Escherichia coli* Rosetta (DE3) separately. After the bacterial culture reached an optical density (OD) of 0.6–0.8 at 600 nm in LB at 37°C, the protein expression was induced at 16°C for ~24 h using 0.2 mM isopropyl-β-d-1-thiogalactoside. Bacteria pellets were harvested by centrifugation, resuspended in phosphate-buffered saline (PBS), and lysed by sonicating on ice. Cell debris was removed by centrifugation (12,000 × g for 1 h) at 4°C. The His-tagged recombinant protein was purified from the obtained supernatant using a 5-ml Ni-HiTrap affinity column (GE Healthcare) and eluted with PBS in the presence of 150–300 mM imidazole. The crude protein was further purified by gel filtration using a Superdex 75 column (GE Healthcare) with PBS. Freshly purified proteins were analyzed via 12% SDS-PAGE, then concentrated to ~2–5 mg/ml, and stored at −80°C for further use. The endotoxin levels in the purified proteins were reduced using Detoxi-Gel Endotoxin Removing Columns (Thermo Scientific, USA) before immunization. Endotoxin levels were quantified using a ToxinSensor Chromogenic Limulus Amebocyte Lysate (LAL) Endotoxin Assay Kit (Genscript, China). Endotoxin contamination levels of all proteins (1 μg/ml) used in this study were under the acceptance level (<0.1 EU/ml).

### Vaccination and Sample Collection

Vaccines were formulated with 50 μg purified proteins, 300 μg Alum (InvivoGen), and 30 μg HPLC-purified TLR9 agonist CpG oligodeoxynucleotide 1826 (CpG ODN1826: 5′-TCCATGACGTTCCTGACGTT-3′; Hua gene) in 200 μl PBS per mouse.

Female BALB/c mice were immunized with the prepared mixtures subcutaneously (s.c.) three times with an interval of 7 or 10 days between each dose. Seven days after the third immunization, sera were obtained from blood samples collected by retro-orbital bleeding technique.

### ELISA for SP-Specific Antibody Assessment

ELISA was performed as described previously (28). Briefly, 96-well plates (Costar) were coated with 1.2 μg of synthetic SP (>90% purity; Sangon Biotech, Shanghai, China) per well and incubated overnight at 4°C. Individual sera were serially diluted from 1:100 to 1:4,096 in a blocking buffer (0.05% Tween, 3% milk in PBS) and incubated for 1 h at 37°C. Subsequently, 100 μl of horseradish peroxidase (HRP) conjugated rabbit anti-mouse antibody subtypes including IgA, IgG, IgG1, IgG2a, IgG2b, or IgG3 (Santa Cruz) at dilutions of 1:2,500 were added, followed by incubation for 1 h at 37°C. The color reaction was developed with TMB (Qiagen) for 30 min and then stopped with 2 M H_2_SO_4_. The absorbance was detected at 450 nm. Sera from PBS- and FD^mut^-treated mice were used as control. Antibody titers were defined as logarithm_10_ of the reciprocal of the highest dilution giving twice the OD of negative control sera (29, 30).

### Splenocyte Proliferation

Seven days or 45 days after the final boost immunization, the spleens of the mice were collected, dissociated into single-cell suspension mechanically, passed through a 70-μm cell strainer (BD Pharmingen), and lysed by red blood cell lysing buffer (139.6 mM NH_4_Cl, 16.96 mM Tris, pH 7.2–7.4). The cells were then re-suspended in RPMI-1640 medium containing 10% FBS, 20 IU/ml IL-2, and 1% P/S, and the concentration was adjusted to 3 × 10^6^ cells/ml; after this,100-μl aliquots were added to a well of 96-well flat plates. Splenocytes were then incubated for 72 h in a cell incubator with (stimulated) or without (unstimulated) stimulation with SP (12 μg/ml). Subsequently, cells were incubated with 10 μl/well in a Cell Counting Kit-8 (CCK-8; Beyotime, China) solution for 2 h at 37°C. Stimulation index (SI) of the splenocytes was determined in triplicate samples by the ratio of the OD of stimulated cells to that of unstimulated at 450 nm (31).

Cell cultures mixed with CCK-8 were used for measuring the OD at 450 nm. Splenocytes isolated from PBS-vaccinated mice served as the negative control in both cell proliferation and cell killing assays.

### Anti-Tumor Activity *in vivo*

For determining the preventive Anti-Tumor effects of the vaccine, the prepared vaccines (antigen + Alum + CpG) were immunized at 10-day intervals three times and tumor model experiments were set 1 week after the third immunization via a subcutaneous injection with a high-dose CT26 cells (3 × 10^5^/per mouse) into the right front flank of female BALB/c mice (*n* = 5–8 per group). In the preventive setting of low-dose CT26 cells, female BALB/c mice (*n* = 5–8 per group) were inoculated with 1 × 10^5^ CT26 cells in the same manner with the same treatment. DTT- or FD^mut^-treated mice served as controls.

To assess the therapeutic effects, mice aged 6–7 weeks were challenged s.c. with 2 × 10^5^ CT26 cells. Two days after the administration of tumor cells, the mice were randomly assigned (*n* = 5–8) into four groups. Two groups were administered with indicated antigens combined with CpG and Alum at 1-week intervals for a total of three times. The remaining two groups were administered with PBS and FD^mut^, and were used as controls.

Tumor development was monitored every 2 or 3 days, and two-dimensional measurements were noted using a Vernier caliper. Tumor size was determined according to the following equation: tumor size = 0.5 × length × (width)^2^. Tumor volumes reaching 2,000 mm^3^ were recorded as death and the mice were sacrificed. Tumor growth and survival curves were drawn and analyzed.

### Fluorescence-Activated Cell Sorting (FACS) Analysis

To elucidate the immune cell profile in tumor-bearing mice, single-cell suspensions of splenocytes and TILs were prepared when the tumor size reached ~1,500 mm^3^. Splenocytes were isolated as described in section Splenocyte Proliferation. For TIL isolation, tumor tissues were pressed though a 70-μm nylon mesh, and a lymphocyte separation medium specific for TILs (Solarbio, China) was used for purification according to the instructions mentioned in the kit.

For cell surface marker staining, antibodies including anti-mouse CD3e-PerCP-Cy™5.5 Hamster (clone 145-2C11), anti-mouse CD4-fluorescein isothiocyanate (FITC) (clone RM4-5), or anti-mouse CD8a-phycoerythrin (PE) (clone 53–6.70) (BD Pharmingen) were added to cell suspensions directly after washing. IFN-γ was detected following the recommended intracellular cytokine staining (ICS) protocol of the Cytofix/Cytoperm solution kit (BD Pharmingen). The Transcription Factor Buffer Set kit (BD Pharmingen) was used for intranuclear protein staining for detecting Foxp3 expression. Allophycocyanin (APC)-conjugated anti-mouse IFN-γ (clone XMG1.2; BD Pharmingen) and PE-conjugated anti-mouse Foxp3 (clone R16-715; BD Pharmingen) were used for IFN-γ and Foxp3 staining, respectively. After staining, the cells were re-suspended in PBS with 2% FBS and analyzed using the CytoFLEX Flow Cytometer (Beckman Coulter). FlowjoV10 software was used for analyzing the collected data.

### Intracellular Cytokine IFN-γ Detection

Bone marrow cells of naïve BALB/c mice were obtained according to the protocol reported by Mayordomo et al. (32) and cultured in a cell culture medium with 20 ng/ml granulocyte-macrophage colony-stimulating factor (GM-CSF) and 20 ng/ml interleukin-4 (IL-4) (Sino Biological, China) for 6 days to generate bone marrow–derived dendritic cells (BMDCs). BMDCs and splenocytes of immunized mice were co-cultured for 48 h at a ratio of 1:10 in a cell culture medium containing SP peptide. Subsequently, 2 μg/ml brefeldin A (BFA) (Multi Science, China) was added to block the IFN-γ transport processes, followed by co-incubation for 6 h. Finally, the cells were collected and stained with fluorescein-labeled antibodies as an ICS procedure. Mice immunized with PBS were used as negative controls.

### Quantitative Real-Time PCR (qRT-PCR) Assay

To identify immune cytokines in tumor tissues, the cDNA of the tumor tissue was generated via a protocol similar to that followed for CT26 cells and then qRT-PCR was performed using an SYBR Green kit (QiaGen, China) to measure the gene expression. The ΔΔCt method was used for data analysis.

### Immunohistochemical (IHC) Analysis

CD8^+^ T-cell subsets in tumor tissues were analyzed by IHC analysis. Concisely, paraffin-embedded blocks of tumor tissues were cut into 3-μm slices and stained with rat anti-mouse CD8α (53–6.7) (Santa Cruz Biotechnology). To assess the primary antibody, an HRP-labeled biotin–streptavidin detection kit (Santa Cruz Biotechnology) was used following the manufacturer's instructions. Fluorescence graphs were captured using a confocal microscope.

### Statistical Analysis

All data were analyzed using GraphPad 7.0 software (San Diego, USA). Results are presented as means ± SD. The statistical significance between two value sets was assessed with two-tailed Student's *t*-test. Anti-Tumor efficacy *in vivo* was compared by Kaplan–Meier analysis and log-rank test. *P* < 0.05 were considered significant.

## Results

### Design and Expression of SP-Based Antigens

Mutant KRAS is a poorly immunogenic target that elicits limited immunogenicity (17). The immunogenicity of self-proteins has been reported enhanced by fusion with DTT (27, 33, 34). We incorporated DTT with mutant KRAS to improve antigen-specific immune responses.

**Figure 1:**
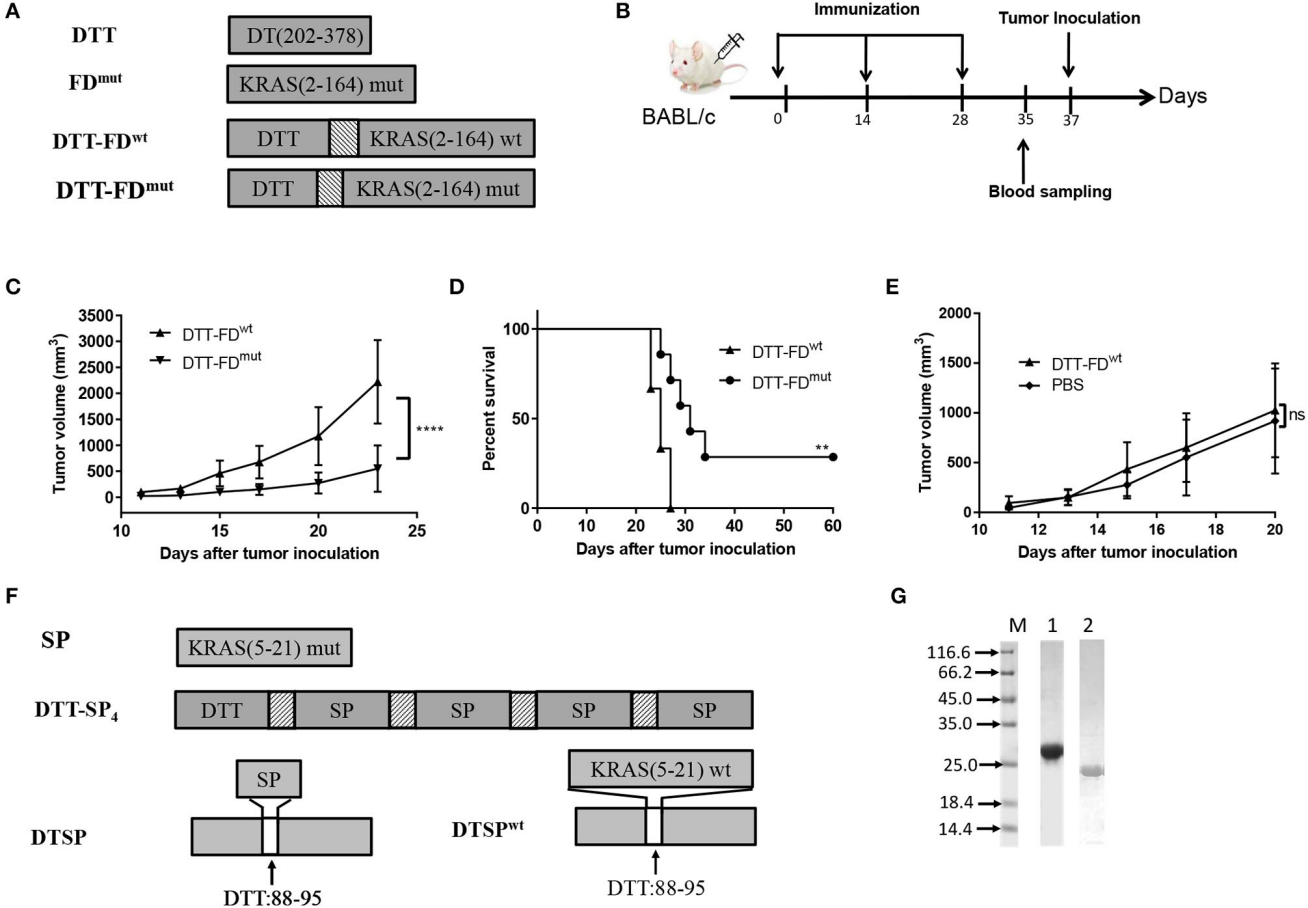
Rationally designed mutant peptide-based vaccines. **(A–E)** Contributions of mutated and non-mutated epitopes to Anti-Tumor efficacy were determined when DTT was used as a carrier protein. **(A)** DTT is the diphtheria toxin T-domain, corresponding to amino acids 202–378 of DT. FD^mut^ represents KRAS G domain (residues 2–164) carrying the G12D mutation. DTT-FD^mut^ and DTT-FD^wt^ were constructed by fusing KRAS G domain (residues 2–164) to the C-terminal of DTT through a GS linker. DTT-FD^mut^ and DTT-FD^wt^ represent presence and absence of KRAS G12D mutation, respectively, in the fusion construct. **(B)** Flow chart of immunization and tumor inoculation. **(C–E)** DTT-FD^mut^, DTT-FD^wt^, and PBS were separately formulated in Alum and CpG. Female BALB/c mice (*n* = 5–8) received the formulated vaccines three times at 2-week intervals. Mice were injected with 2 × 10^5^ cells/mouse 10 days after the last immunization. **(C)** Tumor growth curves of DTT-FD^mut^ and DTT-FD^wt^ were plotted by averaging tumor size over time in each group. Data are presented as means ± SD. ^****^p < 0.0001, Student's *t*-test **(D)** Kaplan–Meier survival curve. ^**^p < 0.01, ns, not significant, log-rank (Mantel–Cox) test for significance. **(E)** Tumor growth curves for DTT-FD^wt^ and PBS. Data are plotted as means ± SD. ns, not significant. **(F,G)** Design and purification of mutant peptide-based vaccines. **(F)** Schematic representation of DTT-SP_4_ and DTSP. SP represents residues 5–21 of KRAS containing the G12D mutation. DTSP^wt^ represents wild-type DTSP lacking the KRAS G12D mutation. The texture box stands for the linker sequence (GG). The white box denotes the position of DTT from 88 to 95 replaced with SP. **(G)** Expression levels of DTT-SP_4_ (lane 1) and DTSP (lane 2).

Whether non-mutant epitopes of KRAS G-domain contribute to Anti-Tumor activity when DTT is used as a carrier protein was uncertain. Therefore, we first constructed DTT-FD^wt^ and DTT-FD^mut^ by fusing the wild type and the mutant type (containing G12D) of KRAS G-domain, respectively, to the C-terminal of DTT via a GS linker (Figure 1A), and assessed their Anti-Tumor efficacies following the vaccination procedure displayed in Figure 1B. Mice administered with DTT-FD^mut^ showed better Anti-Tumor efficacy than those administered with DTT-FD^wt^ in the KRAS G12D mutation containing CT26 tumor model (Figures 1C,D). Furthermore, no significant differences in tumor growth were observed between the DTT-FD^wt^-treated group and PBS-treated control group (Figure 1E). The result suggests that in the presence of DTT, the KRAS mutant epitope shows a higher Anti-Tumor efficacy than any other non-mutated epitope.

Therefore, we further selected the 17-mer KRAS peptide containing G12D mutation (SP) and designed two vaccine forms. The amino acid residues at 88–95 of DTT corresponding to 290–297 of DT has previously been identified as an ideal site for displacement to enhance immune responses of self proteins (27). Therefore, we constructed one antigen by replacing amino acids of 88 to 95 in DTT with SP (named as DTSP) (Figure 1F). For an other antigen, four copies of SP were linked using the GG linker and fused to DTT in tandem (named as DTT-SP_4_) (Figure 1F). The recombined antigens were expressed in *E. coli* system. Purified DTT-SP_4_ appeared to be at ~27 kDa and DTSP approximated to 20 kDa on 12% SDS-PAGE (Figure 1G).

### Both DTT-SP_4_ and DTSP Vaccination Induce SP-Specific Antibody Response and Cellular Response

**Figure 2:**
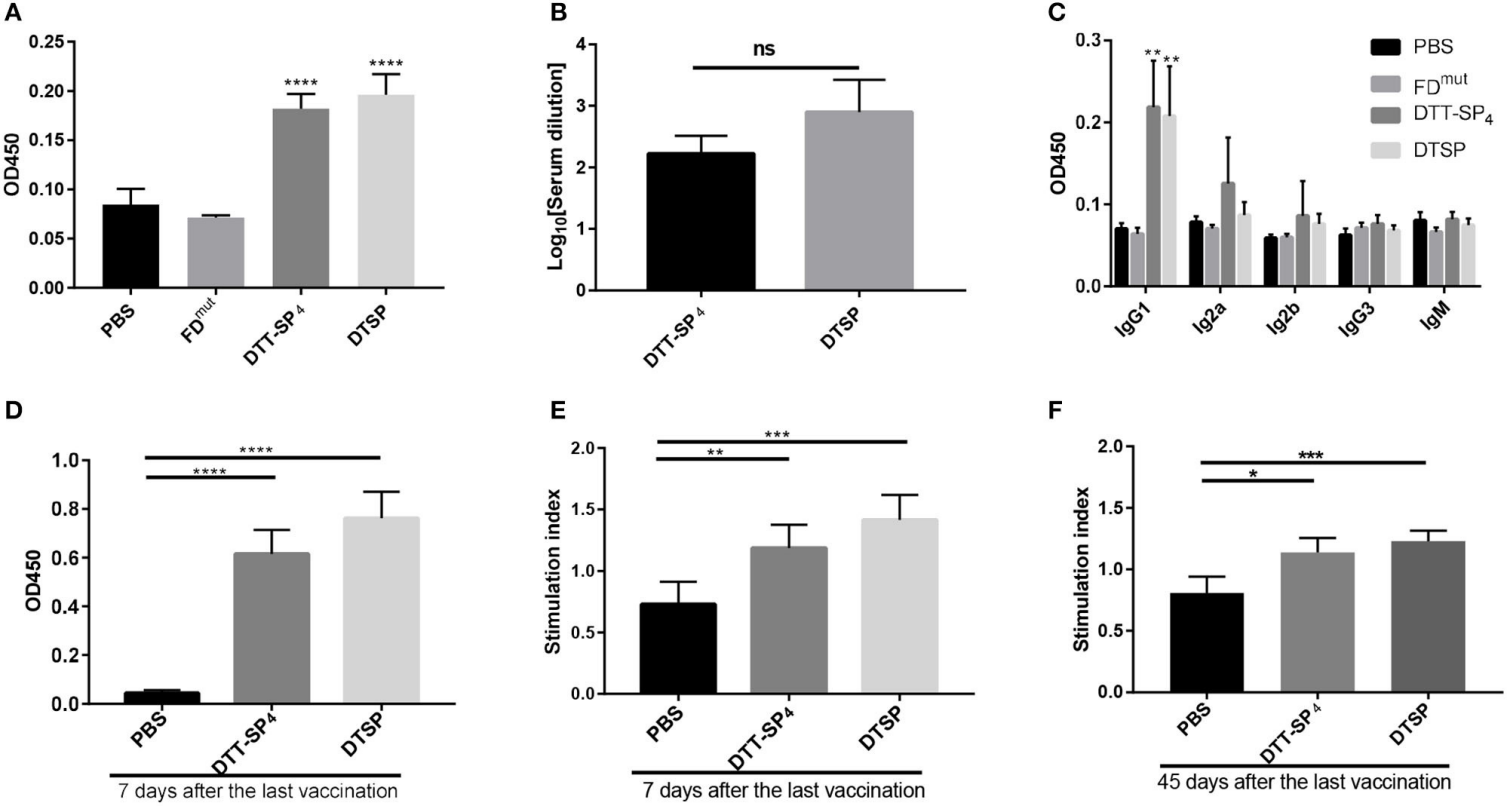
Immunogenicity of recombinant SP antigens. **(A–F)** In all, 6- to 8-week-old female BALB/C mice (*n* = 5) were vaccinated with DTT-SP_4_ or DTSP formulated in Alum and CpG three times at 10-day intervals. **(A–C)** Sera were collected 5 days after the final injection to assess antibody response. **(A)** Total IgG antibody reaction against SP was tested by ELISA. Sera were diluted 1:100. **(B)** Total IgG antibody titers were expressed as logarithm_10_ reciprocals of the highest dilution giving twice the absorbance of the PBS sera. Sera were serially diluted from 1:100 to 1:4,096. **(C)** The level of serum IgG subclass of antibodies in response to SP at a 1:100 dilution. **(D–F)** Splenocytes were harvested 7 days or 45 days after the third immunization and stimulated with SP for 72 h *in vitro*. **(D,E)** Splenocytes were isolated 7 days after the final vaccination. **(D)** The absorbance of stimulated cells at 450 nm in the presence of 10 μL CCK-8 solution. **(E)** SIs were calculated using the ratio of the OD of stimulated cells to that of unstimulated cells at 450 nm. **(F)** Forty-five days after the last vaccination, SI of indicated splenocytes was determined. These data are presented as means ± SD. ^****^p < 0.00001, ^***^p < 0.001, ^**^p < 0.01, ^*^p < 0.05, ns, not significant, Student's *t*-test.

Specific antibody response is correlated with immunogenicity (35). To evaluate whether the recombinant antigens could successfully increase SP immunogenicity, anti-SP IgG antibodies were measured by ELISA on day 35 after the third injection. As shown in Figure 2A, mice immunized with DTT-SP_4_ or DTSP induced higher anti-SP antibody levels than those vaccinated with FD^mut^ or PBS. Antibodies from the PBS- or FD^mut^-treated groups were barely detectable. The average anti-IgG antibody titer in DTSP was slightly higher than that in DTT-SP_4_, but no significant differences were observed (Figure 2B). Conspicuous IgG antibody responses indicated that DTT-SP_4_ or DTSP containing foreign Th epitopes enhanced the immunogenicity of SP. Subtype analysis showed that anti-SP IgG1 antibody levels significantly increased (*p* < 0.01) in DTT-SP_4_ or DTSP immunized mice. Levels of IgG2a and IgG2b subtypes increased to a lesser extent. Both groups showed a uniformly low IgM and IgG3 response (Figure 2C). Subtype reactions suggested that the increased IgG antibody level principally resulted from the increased levels of IgG1, IgG2a, and IgG2b. As a rule, IgG1 levels are associated with T-helper (Th) 2 profile, whereas IgG2a, IgG2b, and IgG3 levels are predominantly associated with Th1 profile (30, 36). The ratios of IgG1 to IgG2a + IgG2b + IgG3 were almost ~1 in DTT-SP_4_- or DTSP-treated groups, revealing a mixed Th1/Th2 response.

To assess the cellular response activated by DTSP or DTT-SP_4_, 7 days after the final immunization, splenocytes were collected and stimulated with SP for 72 h, and cell proliferation was detected using a CCK-8 solution. In comparison with splenocytes from mice treated with PBS, we observed vigorous splenocyte proliferation in DTSP and DTT-SP_4_ groups (Figure 2D). The average SI was highest in DTSP-treated group, with nearly two times the SI observed in the PBS-treated group, and was slightly lower than that in the DTT-SP_4_-treated group (Figure 2E). The same trend in average SI values was also observed 45 days after the last vaccination (Figure 2F). The proliferation and SI data suggest that vaccination with either DTSP or DTT-SP_4_ can elicit SP-specific memory lymphocyte responses.

### DTT-SP_4_ or DTSP Vaccination Offers a Protective Effect Against Tumor Development in the CT26 Tumor Model

**Figure 3:**
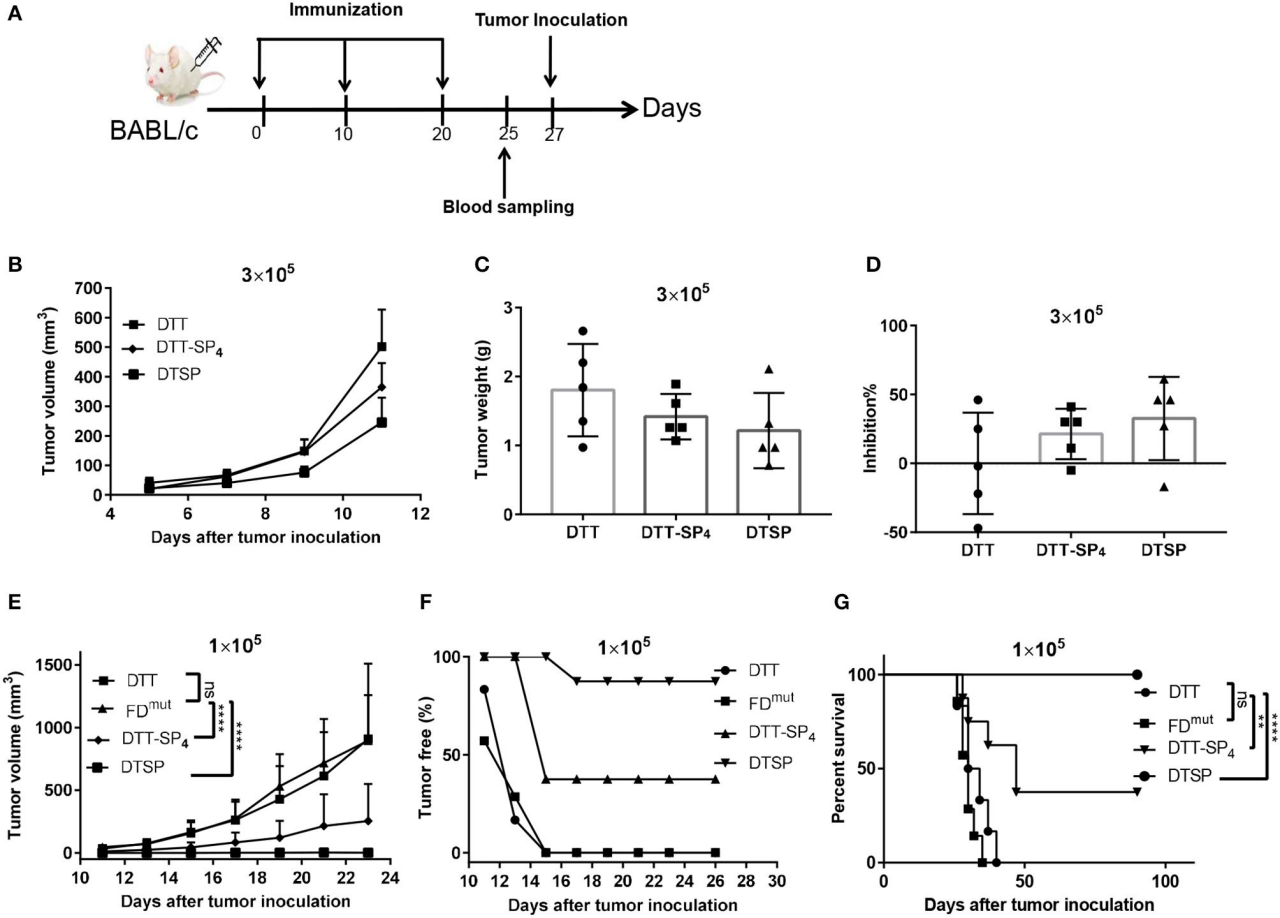
Protective effect of DTT-SP_4_ or DTSP against tumor development after receiving two different CT26 cell doses in the tumor model. **(A)** Flow chart of immunization and tumor inoculation. **(B–G)** Mice were immunized with Alum and CpG formulated DTSP, DTT-SP_4_, FD^mut^, or DTT, three times at 10-day intervals. Mice received two different doses of CT26 cells s.c. into the right flank 1 week after the third administration of the vaccine. **(B–D)** Female BALB/c mice (*n* = 5–8) were inoculated with 3 × 10^5^ CT26 cells/mouse. **(B)** Tumor volumes were determined in individual mice and calculated for each group. Data are means ± SD. Student's *t*-test. Mice were sacrificed on day 23 after tumor challenge. Tumors weights were measured **(C)** and the tumor inhibition rates were evaluated **(D)**. **(E–G)** Immunized mice were challenged with CT26 cells (1 × 10^5^ cells/mouse). **(E)** Tumor growths were monitored every 2–3 days after the tumors were palpable and were presented as tumor volume (mm^3^). Data are means ± SD. ns, not significant, ^****^p < 0.0001, ns, not significant, Student's *t*-test. **(F)** The proportion of tumor-free mice was plotted at different time points after tumor injection. **(G)** Percent survival was plotted by Kaplan–Meier method, and log-rank (Mantel–Cox) test was used to calculated the *p*-value. ^**^p < 0.01, ^****^p < 0.0001, ns, not significant.

Based on the humoral and cellular immune activities of DTT-SP_4_ and DTSP identified *in vitro*, we next assessed whether the two vaccines are capable of preventing tumor development *in vivo*. On day 35 after receiving three doses of the indicated vaccines at 10-day intervals, the mice were, respectively, injected with high-dose and low-dose tumor cells to establish two different preventive models (Figure 3A).

With a high tumor cell dose (3 × 10^5^ cells/mouse), all mice rapidly developed a tumor on day 3. On day 11, the average tumor size was significantly smaller in DTSP-treated group than in the DTT control group (*p* = 0.0028); and the average tumor size in DTT-SP_4_-treated group was smaller than that in the DTT control group as well, but the difference was less significant (*p* = 0.0407; Figure 3B). At the endpoint, all mice were sacrificed on day 23 after tumor inoculation, and the tumor tissues were harvested and weighed. The mean tumor weights in the DTT-SP_4_-treated group (1.42 ± 0.33 g) and DTSP-treated group (1.22 ± 0.55 g) were lower than that in the DTT control group (2.01 ± 0.56 g) (Figure 3C). The average tumor inhibition rate was found to be 32.60% in the DTSP-treated group but only 21.40% in the DTT-SP_4_-treated group (Figure 3D). Taken together, both DTT-SP_4_ and DTSP have Anti-Tumor effects in a high-tumor dose model, but the efficacy is limited.

DTT-SP_4_ and DTSP both showed striking Anti-Tumor efficacies with a lower tumor dose (1 × 10^5^ cells/mouse). We observed slowed tumor growths in DTT-SP_4_-treated and DTSP-treated groups than in DTT control group; particularly, the average tumor size after DTSP treatment was below 50 mm^3^ even on day 23. No significant growth difference was observed between the DTT control group and FD^mut^ group (Figure 3E and Supplementary Figure S1A). Moreover, on day 13, all DTT-SP_4_-treated or DTSP-treated mice were tumor free, whereas only 16% of DTT-treated mice and 28.5% of FD^mut^-treated mice were tumor free. Further, all DTT- or FD^mut^-treated mice developed tumors on day 15. In contrast, 37.5 and 87.5% of mice treated with DTT-SP_4_ and DTSP, respectively, remained tumor free after 90 days of tumor inoculation (Figure 3F). Furthermore, the median survival time of mice treated with DTT-SP_4_ increased by 15 days compared with that of mice treated with DTT. More remarkably, the survival percentage was 100% on Day 90 and overall survival duration was significantly prolonged in DTSP-treated mice compared with those in control mice. Notably, one tumor-bearing mouse in DTSP-treated group survived for more than 90 days. Consistent with the growth curve findings, there was no significant difference in the median survival duration between DTT-treated mice and FD^mut^-treated mice (Figure 3G).

These data indicate that DTT-SP_4_ and DTSP can protect mice from tumor growth, and that especially at low-tumor dose, the tumor inhibition rate after DTSP treatment is 100%.

### DTT-SP_4_ or DTSP Vaccination Exerts a Therapeutic Effect in a CT26 Therapeutic Tumor Model

**Figure 4:**
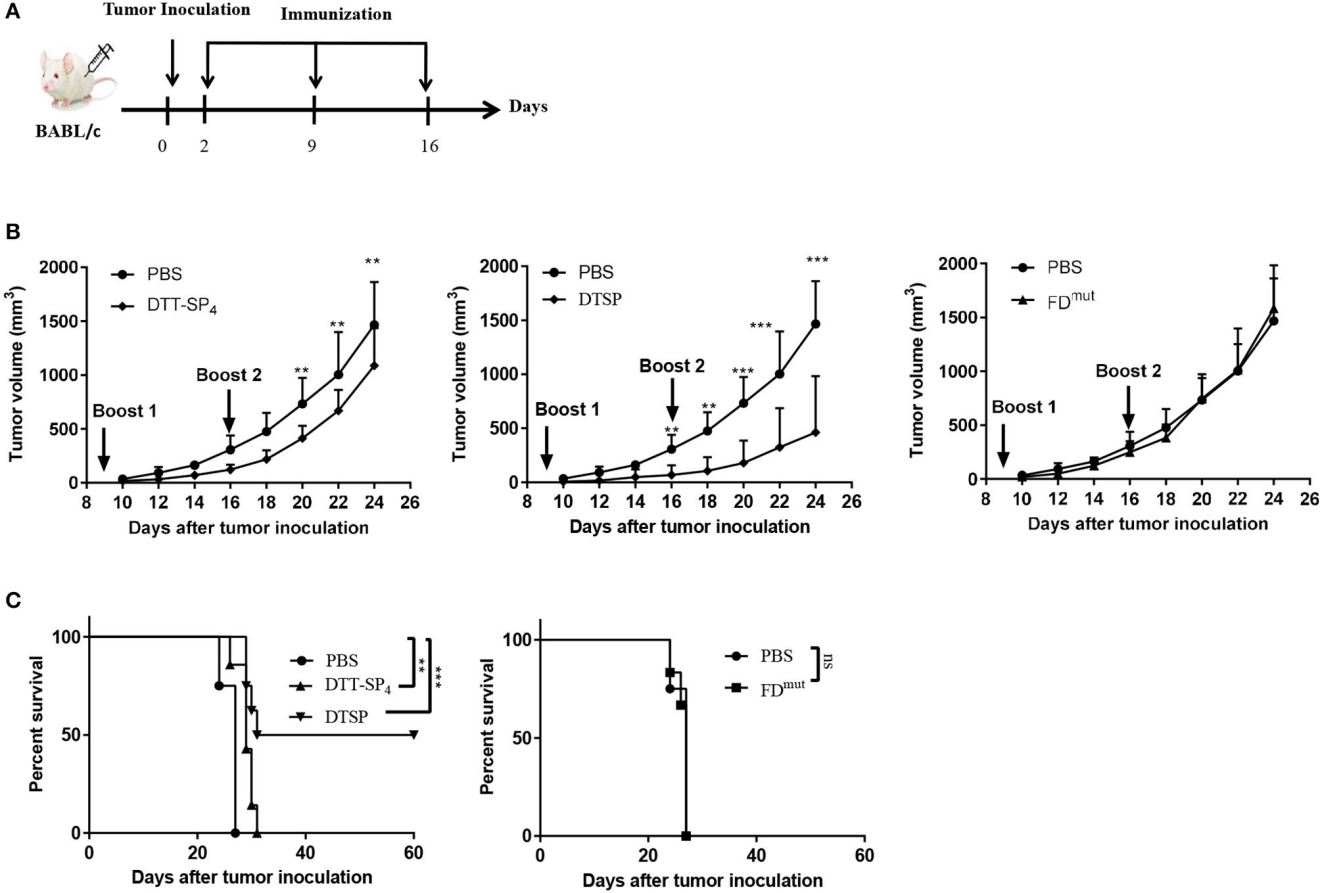
Therapeutic efficacy of DTSP and DTT-SP_4_ vaccination in CT26 tumor model. **(A)** The time course of tumor injections and DTSP or DTT-SP_4_ treatments. **(B,C)** Female BALB/c aged 6–8 weeks were challenged with CT26 cells (1 × 10^5^ cells/mouse) s.c. into the right flank. Two days after CT26 injection, the mice were pooled and assigned randomly (*n* = 5–8). DTSP, DTT-SP_4_, FD^mut^, or PBS formulated in Alum and CpG were administrated to the mice in the respective groups three times at 1-week interval. Tumors were monitored every 2–3 days and measured using a Vernier caliper. **(B)** Tumor growth curves were plotted by measuring tumor volume over time in each group. Data are presented as means ± SD. ^**^p < 0.01, ^***^p < 0.001, Student's *t*-test. **(C)** Kaplan–Meier survival curve. ^*^p < 0.05, ^**^p < 0.01, ^***^p < 0.001, ns, not significant, log-rank (Mantel–Cox) test for significance.

As we observed a significant Anti-Tumor effect in the low-dose preventive CT26 tumor model, we next investigated whether DTT-SP_4_ or DTSP vaccination also displays Anti-Tumor effects against established CT26 tumors. Accordingly, 6- to 8-week-old female BALB/c mice received 2 × 10^5^ CT26 cells/mouse, and 2 days later, the mice were administered with the indicated vaccines three times every 7 days (Figure 4A). Four days after the second booster, the average tumor volume in DTT-SP_4_-treated group was found to be significantly smaller than that in the PBS control group (Figure 4B, left), whereas the average tumor volume after DTSP treatment rapidly showed a difference before the second booster (Figure 4B, middle). There was no difference in tumor growth between FD^mut^ and PBS groups, which is similar to the prophylactic vaccination survival curve findings between the two groups (Figures 4B,C and Supplementary Figure S1B). Both DTT-SP_4_ and DTSP treatments could prolong the median survival duration compared with the two control groups. Strikingly, we observed that 50% of the mice in the DTSP-treated group remained tumor free until 60 days after tumor inoculation at the end of the experiment (Figure 4C). The other 50% of the mice in the DTSP -treated group lived 4 days (day 31) longer than in the two control groups (day 27). These findings indicate that DTSP, although containing only one copy of SP, can still provides a significant therapeutic effect on CT26 tumors, consistent with the preventive effect results on CT26 tumors.

### DTT-SP_4_ or DTSP Vaccination Induces an Antigen-Specific Th1 Response

**Figure 5:**
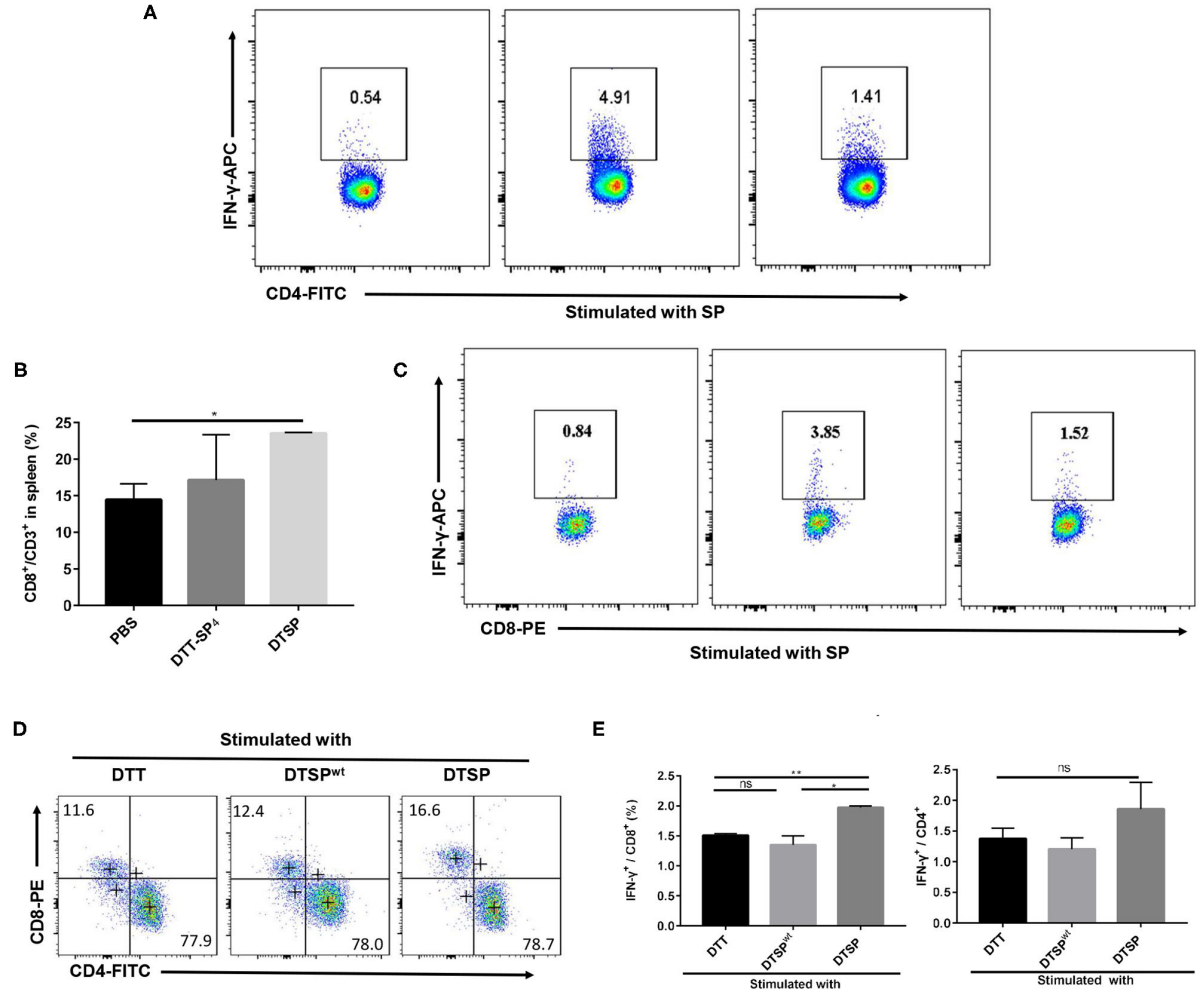
Expression of intracellular antigen-specific IFN-γ in CD4^+^ or CD8^+^ T cells. **(A–E)** Mice were vaccinated with PBS, DTT-SP_4_, or DTSP in combination with Alum and CpG three times at 10-day intervals. Splenocytes (*n* = 3) were harvested 7 days after the final immunization. Splenocytes isolated from immunized mice were co-incubated with BMDCs pulsed with indicated antigens for 48 h, and blocked by BFA for another 6 h. Cells were collected and stained with anti-CD4-FITC, anti-CD8-PE, and anti-CD3-PercP5.5 antibodies. After fixation and permeabilization, cells were stained with anti-IFN-γ-APC antibody and read by flow cytometry. **(A–C)** Splenocytes isolated from PBS-, DTT-SP_4_-, or DTSP-treated mice were pulsed with 12 μg/ml SP. CD8-positive cells **(B)** were gated around CD3^+^ T cells. Data are presented as means ± SD. ^*^p < 0.05, Student's *t*-test. Representative IFN-γ-positive cells in CD4^+^ T cells **(A)** and in CD8^+^ T cells **(C)** are shown. **(D,E)** Splenocytes of DTSP-treated mice were stimulated with 50 μg/ml DTT, DTSP, or DTSP^wt^ *ex vivo*. **(D)** The proportions of CD4^+^ T cells and CD8^+^ T cells in CD3^+^ T cells after stimulation are shown. **(E)** Percentages of IFN-γ-producing CD4^+^ (right panel) or IFN-γ-producing CD8^+^ (left panel) T cells after treatment. These data are presented as means ± SD. ^*^p < 0.05, ^**^p < 0.01, ns, not significant, Student's *t*-test.

IFN-γ, as a typical Th1 cytokine, plays a vital role in the Anti-Tumor activities (37); therefore, we assessed the expression of SP-specific IFN-γ in CD4^+^ T and CD8^+^ T cells isolated from DTT-SP_4_- or DTSP-immunized mice after re-stimulation with SP *in vitro*. The proportion of CD4^+^ IFN-γ^+^ T cells in DTT-SP_4_- and DTSP-treated groups increased to 4.91% and 1.41%, respectively, which were significantly higher than the increase observed in the PBS control group (0.54%) (Figure 5A). Meanwhile, in comparison with the PBS control group, the DTSP-treated group showed and increased ratio of CD8^+^/CD3^+^ T cells; however, no obvious increase was observed in DTT-SP_4_ group (Figure 5B). In addition, 1.52% of CD8^+^ T cells in the DTSP-treated group and 3.85% in the DTT-SP_4_-treated group expressed IFN-γ, only 0.84% of CD8^+^ T cells were found to express IFN-γ in the PBS control group (Figure 5C).

To further illustrate that the DTSP vaccination can elicit a G12D mutation-specific Th1 response, splenocytes isolated from DTSP-vaccinated mice were re-stimulated with DTT, DTSP, or DTSP^wt^. In the DTSP re-stimulated group, the ratio of CD8^+^/CD3^+^ T cells increased (Figure 5D); moreover, the proportion of IFN-γ-producing CD8^+^ T cells increased to 1.97 ± 0.05% (Figure 5E, left), while the groups stimulated with DTSP^wt^ or DTT showed no significant difference. The proportion of IFN-γ^+^-producing CD4^+^ T cells in the group re-stimulated with DTSP was slightly higher than that in the other two groups (Figure 5E, right), but neither the proportion of CD4^+^ CD3^+^ T cells nor the proportion of IFN-γ^+^-producing CD4^+^ T cells showed any significant differences among the three groups (Figures 5D,E). These data demonstrate that DTT-SP_4_ or DTSP vaccination elicits SP-specific IFN-γ expression in both CD4^+^ and CD8^+^ T cells, and that DTSP treatment can induce G12D mutation-specific IFN-γ expression in CD8^+^ T cells.

### DTT-SP_4_ or DTSP Vaccination Increases the Population of CD8^+^ T Cells and Reduces the Proportion of Foxp3^+^/CD4^+^ T Cells in Spleen Tissues as Well as Tumor Tissues of Tumor-Bearing Mice

**Figure 6:**
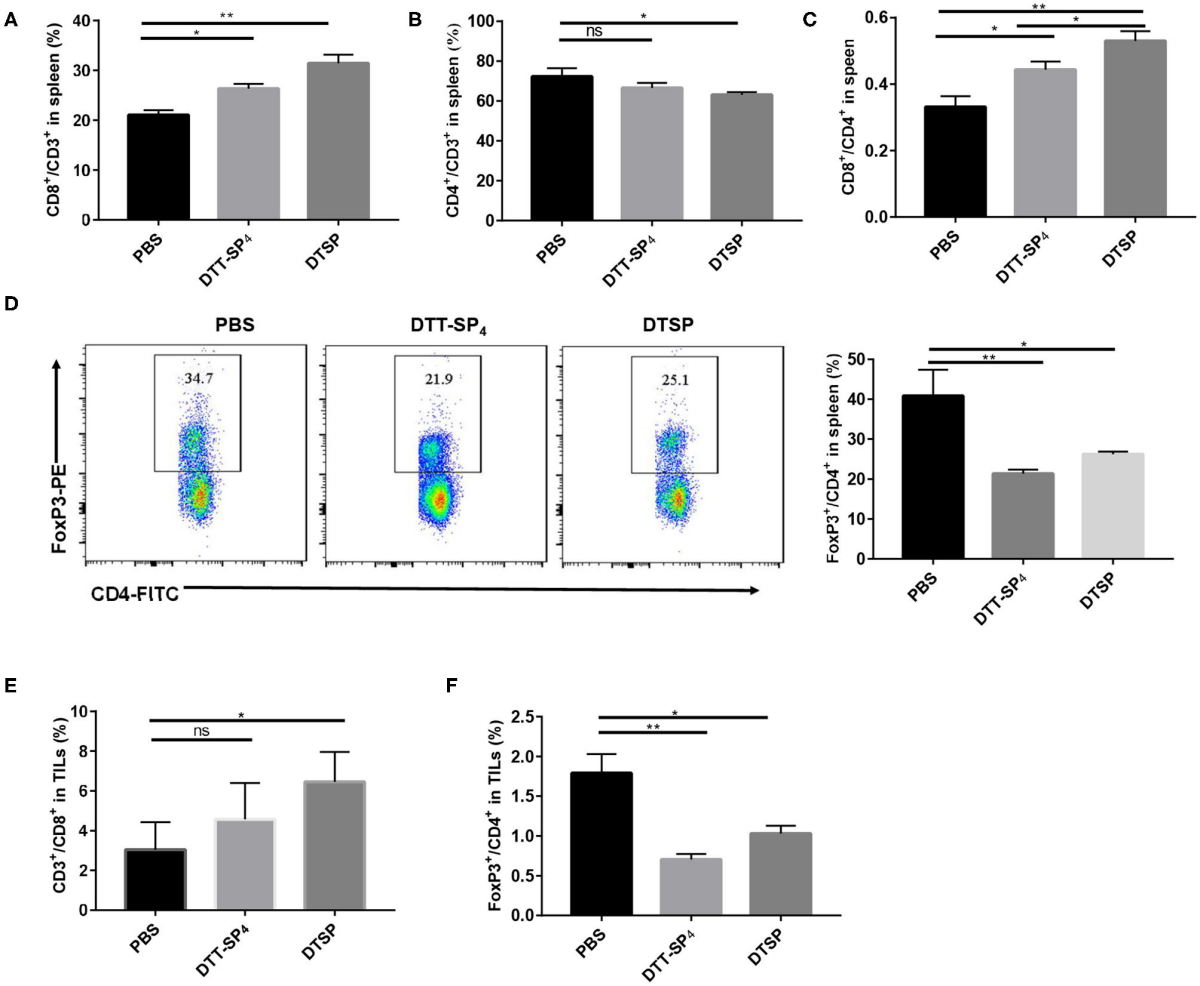
Distribution of CD4^+^ T cells, CD8^+^ T cells, and CD4^+^Foxp3^+^ cells in spleens or TILs of tumor-bearing mice. Mice (*n* = 3) were sacrificed when tumor sizes reached nearly 1,000–1,500 mm^3^. **(A–F)** Splenocytes isolated from the control, DTT-SP_4_-treated, or DTSP-treated group were divided into two tubes at 1 × 10^6^ cells/tube and then stained. One tube was stained with anti-CD4-FITC, anti-CD8-PE, and anti-CD3-PercP5.5 antibodies. The other tube was stained with anti-CD4-FITC and anti-CD3-APC antibodies and further stained with anti-Foxp3-PE antibody after fixation and permeabilization. **(A)** The proportion of CD8^+^/CD3^+^ T cells. **(B)** The proportion of CD4^+^/CD3^+^ T cells. **(C)** Ratios of CD8^+^ T cells to CD4^+^ T cells. **(D)** The proportion of Foxp3^+^/CD4^+^ T cells. **(E,F)** Freshly isolated TILs were divided into two tubes with 1 × 10^6^ cells/tube and then stained following the procedure followed for splenocyte staining. **(E)** The proportion of CD8^+^CD3^+^ T cells. **(F)** The percentage of Foxp3^+^ CD4^+^ T cells. Data are presented as means ± SD. ^*^p < 0.05, ^**^p < 0.01, ns, not significant, Student's *t*-test.

To further clarify Anti-Tumor mechanisms underlying DTT-SP_4_ and DTSP vaccination, we analyzed the subpopulations of T cells in splenocytes and TILs. In the spleen, the proportion of CD8^+^/CD3^+^ T cells dramatically increased to 31.40 ± 1.74% in the DTSP-treated group and slightly increased to 26.30 ± 0.99% in the DTT-SP_4_-treated group; both were higher than that in the PBS control group (21.05 ± 0.92%) (Figure 6A). Conversely, we could only observe a slight decrease in the proportion of CD4^+^/CD3^+^ T cells in the DTSP group, and no significant difference between DTSP and PBS control groups was observed (Figure 6B). Treatment with DTSP and DTT-SP_4_ boosted the ratios of CD8^+^ to CD4^+^ T cells in both the DTSP-treated and DTT-SP_4_-treated groups, respectively (Figure 6C). Further analysis revealed that proportions of Foxp3^+^/CD4^+^ T cells in the DTT-SP_4_-treated and DTSP-treated groups decreased to 21.40 ± 0.95% and 26.27 ± 0.63%, respectively, which are much lower than the ratio in the PBS control group (40.90 ± 6.51%) (Figure 6D).

Similarly, in TILs, the proportion of CD8^+^/CD3^+^ T cells was 6.45 ± 1.51% in the DTSP group, which was almost double compared with the control group value (3.05 ± 1.31%) (Figure 6E). In contrast, DTT-SP_4_ treatment did not remarkably increase the proportion of CD8^+^/CD3^+^ T cells (Figure 6E). IHC staining of tumor tissues with CD8^+^ antibody also showed a similar trend for CD8^+^ T cells among the three groups (Supplementary Figure S2). In addition, the ratios of Foxp3^+^/CD4^+^ T cells in the DTT-SP_4_ and DTSP groups decreased (Figure 6F).

These data demonstrate that DTT-SP_4_ or DTSP vaccination can alter the immune cell subsets both in the spleen and the tumor tissue. It is noteworthy that compared with the DTT-SP_4_-treated group, the DTSP-treated group showed more significant increments in effector cells and reductions in immunosuppressive cells in the tumor tissue and spleen. This was consistent with the fact that the DTSP group showed better Anti-Tumor effects.

### DTSP Vaccination Alters the Immune Cytokine Expression Levels in the Tumor Microenvironment

**Figure 7:**
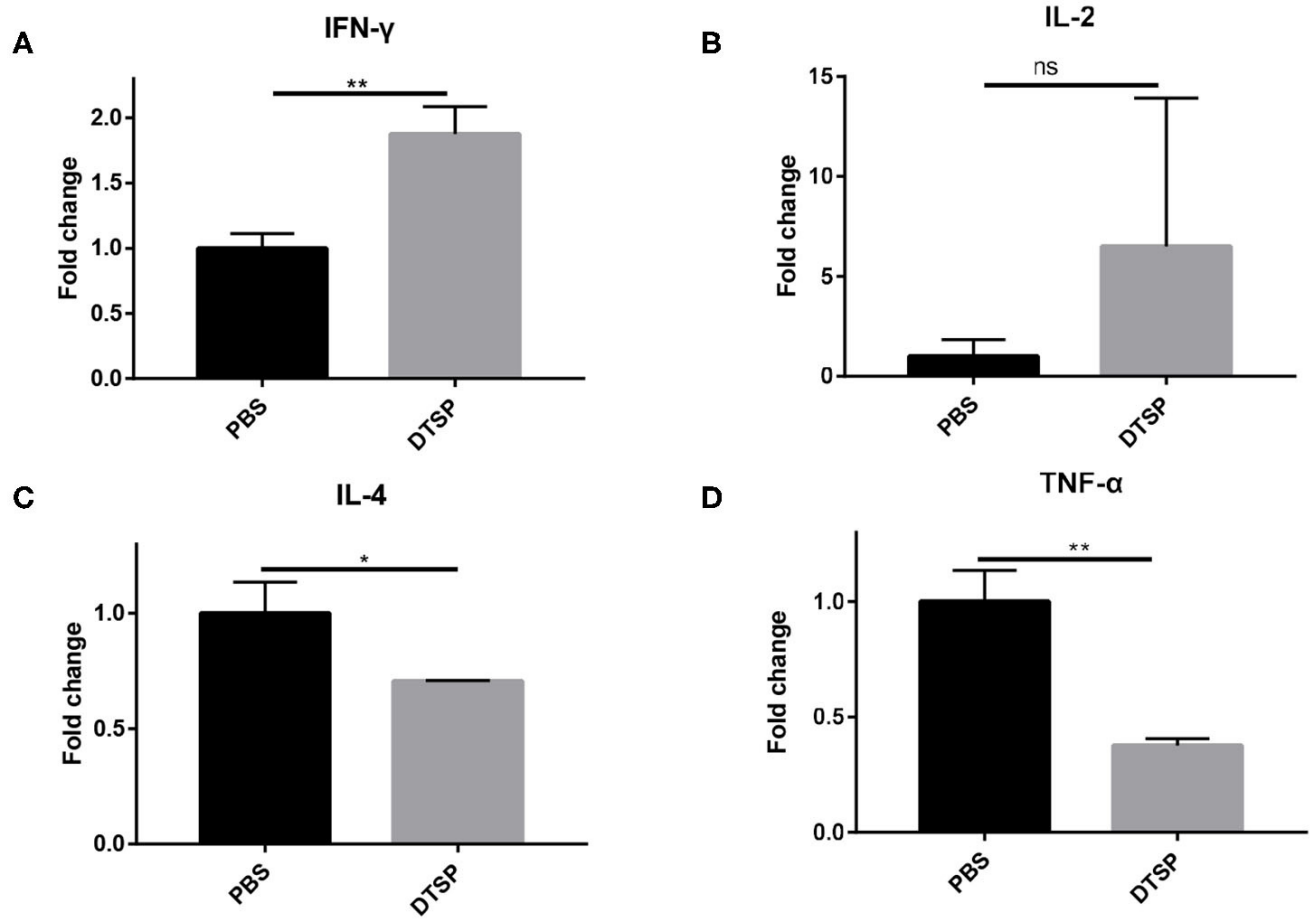
Relative mRNA expression levels of cytokines in tumor tissues. Mice were sacrificed when tumor sizes reached nearly 1,000–1,500 mm^3^; tumor tissues were collected and used for later mRNA expression determination. mRNA expression levels of **(A)** IFN-γ, **(B)** IL-2, **(C)** IL-4, and **(D)** tumor TNF-α were detected via real-time PCR. Relative mRNA expression in the DTSP-treated group was normalized to that in the PBS control group. Data are presented as means ± SD. ^*^p < 0.05; ^**^p < 0.01; ns, not significant, Student's *t*-test.

To further elucidate whether the Anti-Tumor efficacy of DTSP-treated group is associated with alterations in the tumor immune microenvironment, mRNA expression levels of IFN-γ, IL-2, IL-4, and tumor necrosis factor-α (TNF-α) were analyzed. As shown in Figure 7, the level of Th1-related cytokine IFN-γ increased nearly two-fold in the DTSP group compared with that in the PBS control group (Figure 7A). IL-2 level in the DTSP-treated group was also higher than that in the PBS control group but with no significant difference (Figure 7B). In contrast, Th2-related cytokine IL-4 and inflammatory factor TNF-α levels were significantly decreased in the DTSP group compared with those in the PBS control group (Figures 7C,D). TNF-α may be massively expressed by cancer cells in the tumor microenvironment and to a lesser extent by Th1 cells (38): a higher proportion of tumor cells in the PBS control group showed increased mRNA levels of TNF-α in the tumor microenvironment. The higher expression levels of IFN-γ and IL-2 and the lower expression levels of IL-4 in the tumor tissue of DTSP-treated mice suggest that a Th1 immune response but not a Th2 immune response was activated by DTSP treatment. These data combined with the results of IFN-γ expression *in vitro* and the T-cell alterations both in the spleens and TILs described previously suggest that DTSP exerts Anti-Tumor effects mainly by the activation of a Th1 immune response.

## Discussion

The development of a drug that directly targets KRAS G12D mutation or other KRAS mutations remains challenging (8). The smooth surface of the KRAS molecule and the high affinity of KRAS and GTP lead to the difficulty in binding of small-molecule drugs (9, 39). T cells can recognize intracellular mutated peptides displayed on the cell surface by MHC I molecules and are capable of killing tumor cells, and thus inhibit tumor growth (40). An *in vivo* Anti-Tumor effect can theoretically be achieved by directly targeting KRAS mutations through immunotherapies, as long as KRAS mutations are displayed on tumor cells (25). Thus, immunotherapy makes it possible to directly target KRAS mutations without relying on the binding of intracellular KRAS molecule.

Numerous studies have focused on mutant KRAS peptides and proved the safety of peptide vaccines. However, the immune responses among these studies vary, with several studies displaying weak immune responses and even no immune response to KRAS vaccines, although patients display corresponding MHC I expression (17, 25). In the present study, we adopted a feasible approach to enhance the immune response to a mutant KRAS peptide by introducing foreign Th epitopes. DTT has been proven to be a safe carrier protein or scaffold for vaccine developments. DTT contains four universal Th epitopes (aa 69–88, 119–138, 129–148, and 149–168) (41), which could enhance the immunogenicity of self-antigen proteins (27, 33). Therefore, we constructed DTT-SP_4_ and DTSP by fusing the KRAS G12D peptide with DTT to enhance SP-specific immune responses. Alum, an approved adjuvant for human use, was selected in our vaccine formulation, facilitating a slow and sustained release. Alum is known to stimulate Th2 immune responses, but lacks cell-mediated immune stimulation (42). However, for an ideal KRAS G12D cancer vaccine, activated Th1 immune responses are crucial. Previous studies have shown that with the addition of the TLR9 agonist CpG, Alum formulated vaccines can induce a significant Th1 immune response and preferentially increase the proportion of CD8^+^ T cells over that of Tregs (33, 43). Consistent with the reported results, our designed fusion antigens of DTT-SP_4_ or DTSP formulated in Alum and CpG could successfully induce SP-specific antibody and cellular responses (Figure 2). Importantly, preventive and therapeutic Anti-Tumor effects of these were observed in our CT26 tumor model. In addition, the levels of IFN-γ^+^-producing CD4^+^ or CD8^+^ T cells in splenocytes *in vitro* and IL-2 and IFN-γ in tumor tissues increased in the DTSP group. Furthermore, in the spleen and tumor tissues, the populations of CD8^+^ T cells and CD4^+^ Foxp3^+^ T cells were altered after DTSP vaccination. This finding indicates that Alum and CpG formulated DTSP can both exert an Anti-Tumor effect by inducing an SP-specific immune response and alter the composition of T cells in the spleen as well as in tumors to contribute to the Anti-Tumor activity.

Theoretically, for the peptide vaccine, an increase in the copies of the peptide or T- or B-cell epitopes can increase the antigen-specific immune response (44, 45). To our surprise, DTT-SP_4_ with four copies of SP did not show significantly better antibody or cellular responses compared with DTSP with just one copy of SP (Figure 2). Furthermore, the *in vivo* Anti-Tumor response showed that DTSP with a single SP copy was more effective than DTT-SP_4_. This characteristic is consistent with the immune response observed *in vitro*. The functional differences between DTT-SP_4_ and DTSP are particularly intriguing results, and we have no definitive explanation for it. Previous studies have shown that the orientation of epitopes can affect the immune response (45) and peptides with a higher copy number sometimes do not cause stronger immune responses owing to tissue damage (44). Some possible explanations for our observations could be the improper copy number of SP or the improper position of SP in DTT-SP_4_.

Given the significant immune responses induced by DTSP and DTT-SP_4_ *in vitro*, we evaluated their Anti-Tumor efficacies *in vivo*. Castle et al. have previously reported that highly invasive and metastatic KRAS-mutant CT26 cells express functional MHC I molecules (46). The expression of KRAS G12D on CT26 cells was also verified by PCR and sequencing in this study (data not shown). Intracellularly, G12D mutant KRAS should be able to bind to the corresponding MHC I molecules and display on the CT26 cell surface, making it possible for CT26 tumor cells to be lysed by cytotoxic lymphocytes. Recently, Villarreal et al. also used a CT26 model to test the therapeutic effect of *Listeria monocytogenes* (Lm)–based KRAS G12D vaccine (47), which further supports that the CT26 model is appropriate for our study. Compared with DTT-FD^wt^ treatment, DTT-FD^mut^ treatment showed a better tumor inhibition effect in our CT26 preventive model (Figures 1C,D), which not only indicates that mutant KRAS G12D epitope is more important than any other non-mutant epitopes but also suggests that the CT26 model is efficient in detecting the Anti-Tumor activity of DTSP or DTT-SP_4_ containing the KRAS G12D mutant epitope. Recently, several studies have described the tumor-suppressive effects of their KRAS peptide vaccines in different preventive or therapeutic mouse models (47–49). However, to the best of our knowledge, we are the first to confirm that both DTSP and DTT-SP_4_ show certain Anti-Tumor effects not only in a preventive model but also in a therapeutic model. More strikingly, half of DTSP-treated mice were tumor free in the therapeutic model and 87.5% of DTSP-treated mice were tumor free in the low-dose preventive model (Figures 3F, 4B). In both models, DTSP treatment significantly inhibited tumor growths and prolonged survival duration, with the protecting effects lasting for over 60 days, and the mice are still alive when experiment ended (Figures 3E,G, 4B). Notably, the tumor-suppressive effect was also observed in the high-dose prophylactic model; however, tumors rapidly developed into large tumors within a short period of time in this model (Figures 3B–D). A possible explanation is that the high dose tumor cells developed to tumors too rapidly, before the vaccination display therapeutic efficacy or protection. A combination of immune-checkpoint therapies to alter the tumor microenvironment or an expansion of the variety of mutant antigens may be a good improvement, but needs further verification.

In the splenocytes isolated from mice vaccinated with DTT-SP_4_ or DTSP, the proportion of IFN-γ in CD4^+^ and CD8^+^ T cells increased after re-stimulation with SP *in vitro*. We also detected that DTSP vaccination induced mutation-specific IFN-γ secretion. Numerous studies have shown that IFN-γ as a typical Th1 cytokine plays an important role in the early Anti-Tumor response (37), indicating that DTSP may inhibit tumors via a cellular immune response. However, Berner et al. reported that an increased expression of IFN-γ possibly causes CD4^+^ T-cell apoptosis in the secondary stimulation and thus could impair the Anti-Tumor effect (37). Recent studies also show that IFN-γ plays a pro-tumor role by leading CD8^+^ T cells to apoptosis and promoting an immunosuppressive tumor microenvironment during the stage of tumor immunity escape (50). In this study, we also found that although IFN-γ expression level was slightly higher in the DTT-SP_4_-treated group than in the DTSP-treated group after re-stimulating *in vitro* (Figures 3A–D), the Anti-Tumor effect of DTSP was better than that of DTT-SP_4_ *in vivo*. Accordingly, the expression of IFN-γ in the DTT-SP_4_ and DTSP groups *in vitro* did not fully match the Anti-Tumor effects of DTT-SP_4_ and DTSP *in vivo*, which is possibly because of the different levels of immune resistance caused by IFN-γ activation after continuous tumor stimulation. Nevertheless, the obvious expression of IFN-γ in DTSP- or DTT-SP_4_-treated groups after re-stimulation *in vitro* indicates that DTSP or DTT-SP_4_ could induce a successful cellular response. Moreover, the mRNA expression levels of IFN-γ and IL-2 in tumor tissues in the DTSP-treated group were increased, which further supports the hypothesis that the activated Th1 immune response contributes to the Anti-Tumor effect.

According to the statistical results of Cosmic data, G12 mutations account for 83% of all KRAS mutations (26). Among G12 mutations, G12D is a KRAS mutation with the highest frequency, mainly occurring in CRC and PDA (51), and is therefore a meaningful target. Importantly, SP and G-domains of KRAS are 100% homologous between humans and mice; this means that DTSP can directly be translated into a clinical drug. Moreover, in Rosetta cells transformed with a recombinant plasmid containing the DTSP gene, a high amount of expressed soluble DTSP protein can be obtained, reaching 15–20 mg protein per gram of bacterial cells. The expression of DTSP is approximately three times higher than that of DTT-SP_4_ (data not shown). Further, the ease of DTSP protein preparation makes it a potentially cost-effective clinical drug.

In conclusion, our results show that Alum and CpG formulated DTSP, rather than DTT-SP_4_, is more likely to be a preventive and therapeutic clinical drug targeting tumors carrying KRAS G12D mutation.

## Data Availability Statement

The raw data supporting the conclusions of this article will be made available by the authors, without undue reservation, to any qualified researcher.

## Ethics Statement

This animal study was reviewed and approved by Laboratory Animal Ethics and Care Committee of Shanghai Jiao Tong University.

## Author Contributions

YW and RL designed the experiments YW, YZ, PM, GW, and HC performed the experiments. YW wrote the article. All authors read and agree with the article.

## Conflict of Interest

RL was employed by company Shanghai HyCharm Inc. The remaining authors declare that the research was conducted in the absence of any commercial or financial relationships that could be construed as a potential conflict of interest.

## Supplementary Material

Supplementary Figure S1 Tumor growth curve of individual mice in preventive and therapeutic CT26model. **(A)** Tumor growth curve of individual mice in low-dose CT26 model. **(B)** Tumor growth curve of individual mice in therapeutic CT26 model.

Supplementary Figure S2 Tumor infiltration of CD8^+^ T cells in the tumor tissue. Mice from the therapeutic model were sacrificed when tumor size reached nearly 1,000–1,500 mm^3^. **(A)** Representative images of IHC staining for tumor-infiltrating CD8^+^ T cells. **(B)** The density of CD8^+^ T cells were measured by ImageJ. Data are presented as means ± SD. ^***^*p* < 0.001, ^*^*p* < 0.05, Student's *T*-test.
