# Supplementary figures and images for: Recombinant KRAS G12D Protein Vaccines Elicit Significant Anti-Tumor Effects in Mouse CT26 Tumor Models
Source: Front Oncol. 2020 Aug 12;10:1326. doi: 10.3389/fonc.2020.01326 (PMC7435050; doi:10.3389/fonc.2020.01326)

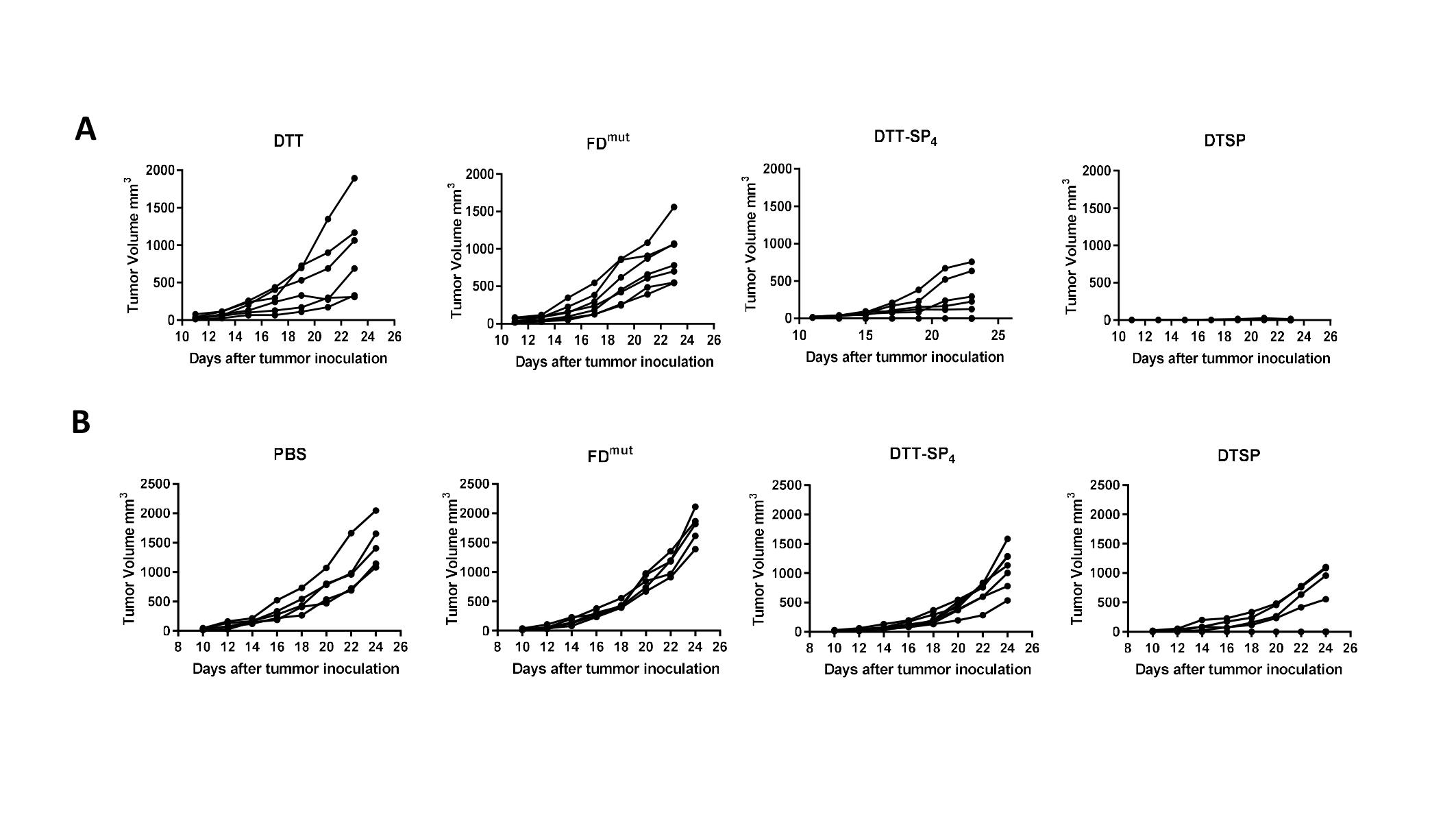

Supplement: Supplementary Figure S1 — Tumor growth curve of individual mice in preventive and therapeutic CT26model. (A) Tumor growth curve of individual mice in low-dose CT26 model. (B) Tumor growth curve of individual mice in therapeutic CT26 model. [file Image_1.TIF]

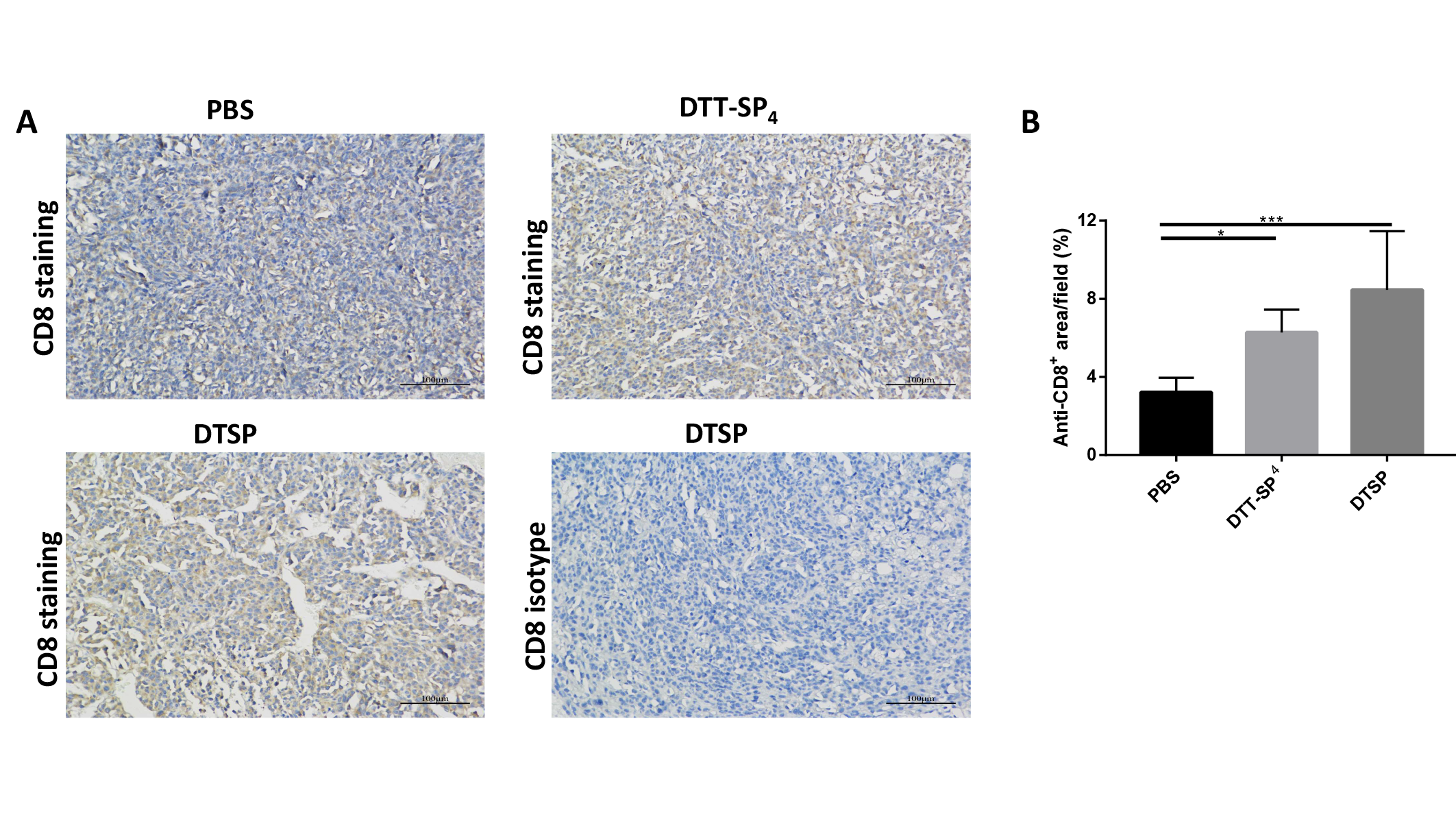

Supplement: Supplementary Figure S2 — Tumor infiltration of CD8+ T cells in the tumor tissue. Mice from the therapeutic model were sacrificed when tumor size reached nearly 1,000–1,500 mm3. (A) Representative images of IHC staining for tumor-infiltrating CD8+ T cells. (B) The density of CD8+ T cells were measured by ImageJ. Data are presented as means ± SD. ***p < 0.001, *p < 0.05, Student's T-test. [file Image_2.TIF]
